# Trends in Total, Added, and Natural Phosphorus Intake in Adult Americans, NHANES 1988–1994 to NHANES 2015–2016

**DOI:** 10.3390/nu13072249

**Published:** 2021-06-29

**Authors:** Kristin Fulgoni, Victor L. Fulgoni

**Affiliations:** Nutrition Impact, LLC, Battle Creek, MI 49014, USA; fulgonik@gmail.com

**Keywords:** dietary phosphorus intake, added phosphorus intake, dietary sources, phosphorus additives

## Abstract

Dietary phosphorus intake in the USA has been consistently greater than the Recommended Daily Allowance (RDA) with several studies reporting associations between intake and health risks as well as all-cause mortality within healthy subjects and patients with chronic kidney disease (CKD). The current study utilized a novel approach to calculate added phosphorus content in foods to determine sources (National Health and Nutrition Examination Survey, NHANES 2001–2016, n = 39,796) and trends in consumption (NHANES 1988–1994, 2001–2016, n = 55,744) of total, naturally occurring, and added phosphorus. Among adults (19+ years), the mean intake of total and natural phosphorus (mg/day) in 1988–1994 as compared with 2015–2016 increased (total: 1292 ± SE 11 vs. 1398 ± SE 17; natural: 1113 ± SE 10 vs. 1243 ± SE 16 mg/day); in contrast, added phosphorus intake decreased during this time (178 ± SE 2.9 vs. 155 ± SE 4.1 mg/day). Added phosphorus as a percent of total ranged from about 14.6% in 1988–1994 to about 11.6% in 2015–2016. The top five sources of total and naturally occurring phosphorus, representing approximately 20% of intake, were cheese, pizza, chicken (whole pieces), reduced-fat milk, and eggs/omelets. The top five sources of added phosphorus were cheese, soft drinks, cakes/pies, rolls/buns, and cookies/brownies, representing 45% of added phosphorus in the diet. Consumption of added phosphorus has decreased over the past few decades, possibly due to increased demand for foods with less additives/ingredients but may also be due to inaccurate phosphorus values in nutrition databases. Further studies are needed to validate the added phosphorus calculations utilized in this study and nutrition databases should consider providing added phosphorus content.

## 1. Introduction

Phosphorus is an essential nutrient that plays multiple roles within the body including energy production, metabolic reactions, and cell structure. The current Recommended Dietary Allowance (RDA) for phosphorus is 700 mg/d for those 19+ years old [1], but the majority of United States citizens consume more phosphorus than the RDA from foods, with the exception of adolescent girls who fall below the RDA [2,3,4,5,6]. The current upper tolerable level (UL) in those 19–70 and 71+ years is 4000 and 3000 mg/d, respectively [1] and according to USDA very few Americans exceed these intakes [7]. Additionally, while these UL are current, they were established in 1997; more recently, the European Food Safety Authority reported an Acceptable Daily Intake (ADI) for phosphates expressed as phosphorus of 40 mg/kg body weight per day which for a 70 kg person equates to 2800 mg/d [8].

In patients with decreased renal function such as individuals with chronic kidney disease (CKD), the dietary phosphorus recommendation is different as compared with those with normal renal function. Management of CKD includes dietary phosphorus restriction and the use of phosphate binder medications. Without proper phosphate excretion, maintenance of phosphorus homeostasis is impacted, leading to hyperphosphatemia. In patients with CKD or those receiving hemodialysis, hyperphosphatemia, defined as serum phosphate levels >4.5 mg/dL [9], has been associated with increased number of cardiovascular hospitalizations, vascular calcification, progression of CKD, death resulting from coronary artery disease, and/or mortality [10,11,12,13,14,15]. While hyperphosphatemia has been a known health risk for CKD patients, recent studies have suggested healthy individuals may also be at risk. Chang and colleagues showed dietary phosphorus intake greater than 1400 mg/day in patients without diabetes, cancer, CKD, or cardiovascular disease (CVD) was associated with all-cause mortality, although this is considerably lower than the ADI established by EFSA [3,8]. Higher serum phosphorus levels in healthy, young adults, were recently shown to be associated with higher likelihood of coronary artery calcification with every 0.5 mg/dL increase in serum phosphorus resulting in a 17% increased risk of higher calcium level categories; however, the authors and others [8] reported numerous limitations of the study (e.g., non-experimental design, small sample size/low power, use of single 24-h recall, etc.) [16]. In patients without CKD and CVD, an increase of 1 mg/dL to serum phosphorus was associated with a CVD hazard ratio of 1.31 [17]. Similarly, a large study of nonchronic kidney disease patients reported an increased risk of end stage renal disease (ESRD), with a hazard ratio of 1.82 for every 0.5 mg/dL increase in serum phosphorus [18]. Findings in the mentioned studies persisted after adjustment for estimated glomerular filtration rate, suggesting associations between high serum phosphorus levels and coronary artery calcification, CVD, and ESRD are independent of renal function.

Phosphate salts are utilized for versatile functions including processing aids (e.g., dairy products), acidulants (e.g., carbonated beverages), flavorants (e.g., processed meats), and leavening agents (e.g., baked goods) [19]. When the nine USDA food groups were subdivided into processed (with additives) and non-processed (without additives), Moore et al. found 22.9% of food servings contained inorganic phosphorus, used in the form of additives [20]. Two previous studies identified foods with phosphorus additives by ingredient lists and measured total phosphorus content alongside a similar grocery item without additives as a reference. The studies showed foods containing phosphorus additives contributed 67–70/100 g of extra phosphorus as compared with foods matched with no phosphorus additives [21,22]. A study that focused on ham found ham with additives, as identified by the presence of phosphate preservatives on the food label, contained 46% more phosphorus than similar products without additives [23]. Simulation of low and high additive/processed diets in 2014 showed high additive diets contributed 606 ± 125 mg more phosphorus per day as compared with a low additive diet [24].

Processed foods generally contain phosphorus additives and are typically more convenient and cost less [22] as compared with natural alternatives. A study, in 2012, found ready-to-eat foods represented approximately 27% of foods reported [25], which likely contributed to increased phosphorus intake through use of additives used for desirable taste/texture profiles and/or shelf stability. Recently, there has been an increased effort to make nutrition information available to consumers on packaging, as well as electronically. However, processed foods attained outside of a grocery store, i.e., fast food, restaurant meals, may not have ingredient lists or nutrition labels, making phosphorus intake estimation and identification of meals with phosphorus additives more challenging. 

There are limited studies on dietary phosphorus quantification broken down into total, added, and natural as well as their respective food sources. Capturing added phosphorus intakes and their trends over time will allow us to understand whether or not phosphate salts significantly impact phosphorus intake and whether intake of phosphorus additives have increased over time. This information would also provide a better understanding of associations between added and natural phosphorus intake and its impact on health. This study aims to analyze phosphorus intake separated into its added and natural sources to further the understanding of phosphorus additives and their impact in the U.S. population and the food supply.

## 2. Materials and Methods

The National Health and Nutrition Examination Survey (NHANES) program is a cross-sectional nationally representative survey of non-institutionalized citizens in the United States. The program includes a dietary interview, What We Eat in America (WWEIA), combined with physical examinations. Data for adults 19+ years from the 1988–1994 and through 2001–2016 NHANES surveys were utilized in the current study. Descriptions have been provided previously for methods and study designs utilized by the NHANES [26,27]. Participants with incomplete or unreliable dietary records as determined by USDA food research staff and those pregnant and/or lactating were removed from the dataset.

**Figure 1:**
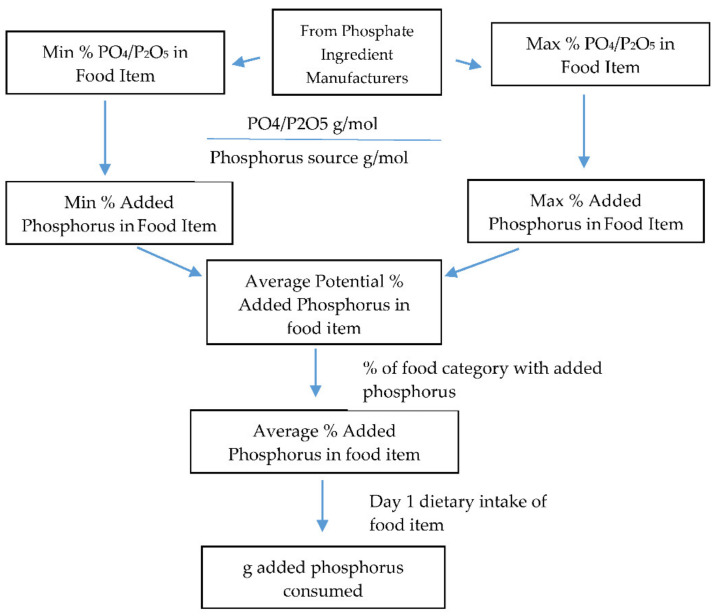
Process of calculating values of added phosphorus in foods and beverages.

Steps used to calculate estimated added phosphorus in food categories are displayed in Figure 1. Information was collected on phosphorus source ingredients and range of levels present in food categories from phosphate ingredient manufacturers (see Supplementary Materials Table S1). The minimum and maximum levels of PO_4_ and P_2_O_5_ were multiplied by their respective molecular weights to obtain the phosphorus weight, and then these weights were averaged for each food category (155). Averages were used since differences between minimum and maximum levels were small, with the largest difference being 0.7% added phosphorus as P_2_O_5_ within food categories white potatoes (baked, boiled, or mashed potatoes) and white potato mixtures. Then, these values were multiplied by the percentage of products in a food category with phosphorus ingredients as determined by an analysis of Innova Market Insights database (see Supplementary Materials Table S2) that contains information on ingredients in foods, to obtain the average percentage of added phosphorus in the food category. Natural phosphorus content was determined by subtracting the calculated added phosphorus from the total phosphorus content provided in the NHANES. Total, natural, and added phosphorus intakes were determined by multiplying the percentage of respective phosphorus in the food item by the Day 1 dietary intake of the food. Summation of total, natural, and added phosphorus from all individual food categories resulted in daily total intake.

One-day dietary recalls were collected using the automated multiple-pass method (AMPM) and used to determine intake trends of total phosphorus, added phosphorus, and naturally occurring phosphorus from all foods and beverages, not including dietary supplements [28], after adjusting for age, gender, and race/ethnicity. Phosphorus sources were determined by utilizing the WWEIA and USDA 155 food categories [29]. Top sources of total, added, and naturally occurring were determined using the population ratio method, as recommended [30].

Least square means, standard errors, and ANOVA were used to assess the difference in intake at each cycle as compared with 1988–1994. Regression analyses were used to assess trends in intake overtime (a) with 1988–1994 designated as 1, and then each cycle of the NHANES as 2–9 for data from 2001–2002 through 2015–2016 providing a regression coefficient depicting change per cycle of the NHANES results, and (b) using the actual year as the independent variable providing a regression coefficient depicting change per year. Analyses were adjusted for age, gender, and race/ethnicity. Analyses were conducted for those 19+, 19–50, and 51+ years for genders combined and for males/females separately. Statistical significance was set at *p* < 0.01 and all analyses utilized SAS 9.4 and survey parameters including strata, primary sampling units, and recommended sampling weights (SAS Institute, Cary, NC, USA).

## 3. Results

### 3.1. Intake and Trends over Time

Dietary intake of total phosphorus and natural phosphorus in 2015–2016 was significantly higher as compared with 1988–1994 at 1398 ± 17 mg/day and 1292 ± 11 mg/day, respectively (Table 1). Males had higher intake of both total phosphorus (males 1612 ± 23, females 1189 ± 16 mg/day) and natural phosphorus (males 1435 ± 22, females 1055 ± 14 mg/day) in 2015–2016 as compared with females. Both total and natural phosphorus dietary intake increased between 1988–1994 and 2015–2016. Total phosphorus intake increased by 5.7 ± 0.6 mg/day per year (*p* < 0.0001) and natural phosphorus intake increased 6.6 ± 0.6 mg/day per year (*p* < 0.0001). Females had a larger magnitude of change for total (males 5.2 ± 0.9, females 6.2 ± 0.6 mg/day per year) and natural phosphorus intake than males (males 6.5 ± 0.9, females 6.8 ± 0.6 mg/day per year).

Intake of added phosphorus (Table 1) in 2015–2016 was 155 ± 4.1 mg/day and significantly less than intake in 1988–1994 (178 ± 2.9 mg/day), decreasing by 0.92 mg/day per year (± SE 0.16 mg, *p* < 0.0001). Females (134 ± 3.4 vs. 147 ± 2.9 mg/day, *p* < 0.0004) and males (177 ± 6.0 vs. 210 ± 4.0 mg/day, *p* < 0.0001) both had significantly lower added phosphorus intake in 2015–2016 as compared with in 1988–1994. The decrease in added phosphorus intake over time was lower in females (−0.61 ± 0.2 mg/day/year) as compared with males (−1.23 ± 0.2 mg/day/year). The percentage of total phosphorus intake attributed to added phosphorus in 2015–2016 was 11.6 ± 0.2% and significantly decreased by 0.14 ± 0.01 percentage units (*p* < 0.0001) yearly since 1988–1994. The percentage of total phosphorus as added phosphorus was similar between males (11.5 ± 0.3%) and females (11.6 ± 0.2%), and the percentage of total phosphorus as added phosphorus decreased similarly in males and females (−0.13 ± 0.01 vs. −0.15 ± 0.01 percentage units per year, respectively).

### 3.2. Intake and Trends over Time by Body Weight Status

When daily total, natural, and added phosphorus intake were also assessed based on mg/kg of body weight, several changes in significance occurred (Table 2). Daily total phosphorus intake in 2015–2016 was 17.5 ± 0.2 mg/kg and was no longer significantly higher as compared with in 1988–1994 (17.7 ± 0.1 mg/kg, *p* = 0.29) and lacked significant cycle and yearly trends. Daily natural phosphorus intake was not significantly higher in 2015–2016 (15.6 ± 0.2 mg/kg) as compared with in 1988–1994 (15.3 ± 0.1 mg/kg, *p* = 0.32), but the increase across the NHANES cycles (0.12 ± 0.02 mg/kg/cycle, *p* < 0.0001) and yearly (0.03 ± 0.01 mg/kg/year, *p* < 0.0001) trends remained significant. Daily added phosphorus intakes for all (1.93 ± 0.04 mg/kg vs. 2.43 ± 0.04 mg/kg, *p* < 0.0001), male (2.03 ± 0.05 mg/kg vs. 2.63 ± 0.05 mg/kg, *p* < 0.0001), and female (1.83 ± 0.05 mg/kg vs. 2.24 ± 0.05 mg/kg, *p* < 0.0001) subjects remained significantly lower in 2015–2016 as compared with in 1988–1994. Significant cycle trends for all (−0.06 ± 0.01 mg/kg/cycle, *p* < 0.0001), males (−0.06 ± 0.01 mg/kg/cycle, *p* < 0.0001), and females (−0.05 ± 0.01 mg/kg/cycle, *p* < 0.0001) persisted.

On a yearly basis between 1988–1994 and 2015–2016, daily added phosphorus intake decreased in all subjects (−0.02 ± 0.002 mg/kg/year, *p* < 0.0001), as well as in males (−0.02 ± 0.003 mg/kg/year, *p* < 0.0001) and females (−0.02 ± 0.003 mg/kg/year, *p* < 0.0001); whereas, daily natural phosphorus intake increased yearly for all (0.03 ± 0.01 mg/kg/year, *p* < 0.0001), male (0.03 ± 0.01 mg/kg/year, *p* < 0.001), and female (0.04 ± 0.01 mg/kg/year, *p* < 0.0001) subjects. Daily total phosphorus intake in mg/kg was not significantly (*p* = 0.016) different over time.

### 3.3. Sources of Phosphorus

Out of the 155 food categories created by the USDA included in the NHANES [29], cheese, pizza, chicken (whole pieces), reduced-fat milk, and eggs/omelets were the top five sources of both total and natural phosphorus and contributed approximately 20% of total and natural phosphorus to the diet (Table 3). The top five sources of added phosphorus contributed approximately 45% of added phosphorus to the diet and were cheese, soft drinks, cakes/pies, rolls/buns, and cookies/brownies.

## 4. Discussion

Multiple studies have addressed dietary phosphorus intake on a total basis, and a few have broken phosphorus into the presence of inorganic and organic. The current study was performed to understand the portions of phosphorus additives and naturally occurring phosphorus contributing to total phosphorus intake. The current study showed total and natural phosphorus intake increased significantly over the past few decades, whereas added phosphorus intake decreased. The increase in total phosphorus over time is in line with a previous study which found a 4% increase in dietary phosphorus from 2001 to 2014 [4].

Female intake of phosphorus showed a slightly higher increase in total and natural phosphorus and a lower decrease in added phosphorus between 1988–1994 and 2015–2016 as compared with males. Previous studies have shown contradicting evidence of gender influence on dietary phosphorus intake and/or serum phosphorus. The 2001–2014 NHANES and Multi-Ethnic Study of Atherosclerosis (MESA) data both show men consumed more phosphorus than women [3,31], but data were not analyzed by body weight status, which may contribute to differences relative to this study. On the other hand, several studies have found females have a tendency to have higher serum phosphorus which may be attributed to dietary intake [16,32,33]. A previous study found a significant linear association between food-additive phosphate and carotid intima-media thickness in females but not in males [34]. A very small study (n = 14) focused on healthy, young females, suggested high phosphorus intake was associated with negative bone metabolism markers [35]. Further studies are required to understand possible health impact differences between genders associated with phosphorus intake.

Several trends in intake of phosphorus changed when body weight status was taken into account. The prevalence of overweight or obese adults increased steadily throughout the years of the current study with an estimated 22.9% of U.S. citizens obese in 1988–1994 and 39.6% in 2015–2016 [36,37]. As body weight increases, the impact of phosphorus intake on the individual decreases on a mg/kg basis. The current study is one of few studies that have taken body weight status into account when assessing phosphorus intake. Further studies are needed to verify the effects of body weight status on total, natural, and added phosphorus intake.

Current intakes of total phosphorus (mean of 1398 ± 17 mg/day) are well below UL and ADI established by IOM and ESFA, respectively. The 99th percentile of the mean for 2015–2016 was about 2097 mg/day which is also well below the UL and ADI established by IOM and ESFA, respectively, further suggesting very few individuals exceed the established UL and ADI from food alone. Added phosphorus, as a percentage of total phosphorus, ranged from about 11 to 14.5% for the NHANES cycles evaluated, similar to that reported by others [8].

Sources of added phosphorus are in-line with phosphorus additives utilized in food processing. The second top source of added phosphorus was soft drinks, which is likely attributed to the utilization of 0.05% phosphoric acid as a flavorant and acidulant in cola beverages [19]. Bakery products, including cakes/pies, rolls/buns, and cookies/brownies, three of the top five sources of added phosphorus, utilize phosphate ingredients for chemical leavening. A 2013 study of top selling grocery products in Ohio found 57% of bread and baked good products contained phosphorus additives [22]. The type and amount of additive varies between products, further increasing the difficultly of quantifying phosphorus intake [19].

Top sources of total and natural phosphorus are similar to results of previous studies that have found meat/poultry/seafood and milk were two of the top five sources of dietary phosphorus [4]. Chicken (whole pieces) was one of the top sources of total and natural phosphorus in this study, but not for added phosphorus. This was unexpected since studies have found 37% of meat, poultry, and fish products [21] and 63% of boneless chicken breast products [38] had added phosphorus present on their labels and were found to have more total phosphorus than non-additive chicken breast [39]. One explanation for this may be the lack of separation between frozen and fresh meat, poultry, and fish products within the NHANES. If these products were separated, it is possible that added phosphorus would be higher due to an increased likelihood of frozen meat, poultry, and fish containing added phosphorus ingredients as compared with fresh products [21]. It is also possible that more recent analyses of poultry products are needed in the NHANES.

Current databases do not separate added phosphorus from total phosphorus and often contain underestimations of total phosphorus content, as well as misclassify and lack product specific data, which may lead to inaccuracies in determining true phosphorus content [5,40,41]. Previous studies in 2007 and 2015 have shown products containing phosphorus additives had higher actual total phosphorus as compared with the estimated amount in nutrition databases [38,42]. A second study, in 2015, on simulated diets with high and low amounts of phosphorus additives showed no significant difference between the database values for the high phosphorus diet estimate and measured [24]. Further delineation of total phosphorus into added and natural phosphorus within databases may help resolve differences observed. High variation in natural and added phosphorus between and among products adds to the complications of estimated total phosphorus intake [20,22,38,42].

The strengths of our study included the use of a national representative sample of non-institutionalized, healthy U.S. citizens, assessment of two metrics of dietary phosphorus intake (mg/day and mg/kg), and fractionation of dietary phosphorus intake into total, natural, and added. The current study has several limitations. The NHANES is an observational study that includes self-reported intakes which are known to be sensitive to under- and overreporting of food [43]. Although the use of covariate sets attempted to remove variables correlated with phosphorus intake, the impact of residual confounding factors with other variables may exist. For example, the study analyzed phosphorus intake by mg/kg to understand whether intake was influenced by body weight, but this metric does not take into account physical activity level nor actual caloric intake. Finally, the method used in this study to calculate added phosphorus in foods is novel and needs further validation in future studies. For full transparency, the base data used for our calculations are present in the Supplementary Materials Tables S1 and S2. While we used an average level of phosphorus additive in data presented here, we additionally, as a sensitivity analysis, estimated added phosphorus intakes assuming that all minimum, and then all maximum, levels of phosphate additives were used. The intakes of added phosphorus for the minimum and maximum use levels of phosphate additives were 141 ± 3.6 and 161 ± 4.1 mg/d, respectively, for 2015–2016, suggesting even at the highest use levels added phosphorus intake was small as compared with the amount occurring naturally. Finally, there may be other sources of added phosphates in foods that are not measured in the NHANES databases that could not be estimated (e.g., lecithin and modified starch) and we were not able to capture all the added phosphorus from mixed dishes where processed cheeses may be used as an ingredient, for example, macaroni and cheese, etc.

The current study found total and natural phosphorus dietary intake increased from 1988–1994 to 2015–2016, while added phosphorus intake decreased during this time. This indicated that increased intake of foods with naturally occurring phosphorus in the USA is the major contributing factor in phosphorus intake. This emphasizes the need for further studies to better understand the role in the diet of added phosphorus intake. Better information on the level of added phosphorus used in foods is needed and such information in the NHANES could be useful for future studies to assess health effects for added phosphorus.

## Figures and Tables

**Table 1:** Total, added, and natural phosphorus mean ^1,2^ intake (mg/day) and percentage of total phosphorus as added for adults 19 years of age and older, the NHANES 1988–1994 and 2001–2016.

Subject Group	NHANES Cycle	Total Phosphorus	Added Phosphorus	Natural Phosphorus	% As Added
All (n = 55,744)	1988–1994	1292 (10.7)	178.1 (2.9)	1113.4 (9.9)	14.6 (0.2)
	2001–2002	1330 (16.1)	192.6 (3.4)	1137.8 (15.9)	15.2 (0.3)
	2003–2004	1324 (16.5)	188.0 (3.2)	1136.2 (15.6)	15.1 (0.2)
	2005–2006	1359 (16.4)	183.6 (4.9)	1175.4 (16.0)	14.2 (0.3)
	2007–2008	1331 (21.2)	174.9 (3.7)	1156.2 (21.1)	13.8 (0.3)
	2009–2010	1417 (11.4)	181.6 (2.6)	1235.4 (10.6)	13.2 (0.2)
	2011–2012	1430 (9.90)	172.3 (3.9)	1257.3 (11.5)	12.5 (0.3)
	2013–2014	1406 (12.4)	175.5 (3.3)	1230.4 (12.7)	13.0 (0.3)
	2015–2016	1398 (17.0)	155.2 (4.1)	1242.7 (15.6)	11.6 (0.2)
	P ^3^	**<0.0001**	**<0.0001**	**<0.0001**	**<0.0001**
	Cycle Trend				
	Beta ^4^	15.06 (1.89)	−3.13 (0.46)	18.19 (1.83)	−0.43 (0.03)
	*p*-value	**<0.0001**	**<0.0001**	**<0.0001**	**<0.0001**
	Yearly Trend				
	Beta ^5^	5.70 (0.65)	−0.92 (0.16)	6.62 (0.62)	−0.14 (0.01)
	*p*-value	**<0.0001**	**<0.0001**	**<0.0001**	**<0.0001**
Males (n = 27,616)	1988–1994	1538 (14.9)	209.9 (4.0)	1328.2 (13.9)	14.4 (0.2)
	2001–2002	1551 (27.3)	219.8 (6.3)	1330.7 (26.3)	14.7 (0.3)
	2003–2004	1546 (23.7)	216.2 (5.2)	1329.8 (22.2)	14.7 (0.3)
	2005–2006	1594 (21.4)	216.3 (6.9)	1377.5 (20.4)	14.3 (0.4)
	2007–2008	1548 (24.9)	198.7 (5.6)	1349.8 (24.5)	13.4 (0.4)
	2009–2010	1656 (16.8)	215.3 (4.7)	1441.0 (15.9)	13.3 (0.3)
	2011–2012	1675 (16.5)	199.4 (5.8)	1475.8 (16.9)	12.4 (0.3)
	2013–2014	1635 (18.1)	200.5 (5.6)	1434.1 (16.8)	12.7 (0.3)
	2015–2016	1612 (23.2)	177.0 (6.0)	1435.3 (21.7)	11.5 (0.3)
	P ^3^	**0.0084**	**<0.0001**	**0.0001**	**<0.0001**
	Cycle Trend				
	Beta ^4^	14.36 (2.80)	−3.95 (0.72)	18.31 (2.66)	−0.40 (0.04)
	*p*-value	**<0.0001**	**<0.0001**	**<0.0001**	**<0.0001**
	Yearly Trend				
	Beta ^5^	5.22 (0.93)	−1.23 (0.25)	6.45 (0.88)	−0.13 (0.01)
	*p*-value	**<0.0001**	**<0.0001**	**<0.0001**	**<0.0001**
Females (n = 28,128)	1988–1994	1052 (10.1)	147.2 (2.9)	905.1 (8.7)	14.8 (0.2)
	2001–2002	1115 (16.8)	165.8 (4.1)	949.5 (15.5)	15.7 (0.4)
	2003–2004	1108 (15.5)	160.5 (3.7)	947.3 (15.7)	15.4 (0.4)
	2005–2006	1130 (17.3)	151.5 (3.8)	978.0 (17.0)	14.2 (0.4)
	2007–2008	1122 (22.0)	151.6 (3.9)	970.3 (21.6)	14.1 (0.4)
	2009–2010	1185 (12.4)	148.9 (2.4)	1036.0 (12.1)	13.1 (0.2)
	2011–2012	1191 (12.8)	146.0 (4.1)	1044.8 (12.0)	12.7 (0.3)
	2013–2014	1185 (13.4)	151.4 (3.3)	1033.4 (14.4)	13.3 (0.3)
	2015–2016	1189 (16.0)	134.0 (3.4)	1055.4 (14.3)	11.6 (0.2)
	P ^3^	**<0.0001**	**0.0004**	**<0.0001**	**<0.0001**
	Cycle Trend				
	Beta ^4^	15.91 (1.87)	−2.30 (0.45)	18.21 (1.77)	−0.46 (0.04)
	*p*-value	**<0.0001**	**<0.0001**	**<0.0001**	**<0.0001**
	Yearly Trend				
	Beta ^5^	6.21 (0.63)	−0.61 (0.16)	6.82 (0.59)	−0.15 (0.01)
	*p*-value	**<0.0001**	**<0.0001**	**<0.0001**	**<0.0001**

^1^ Results adjusted for age, gender, and ethnicity; ^2^ values are represented as mean (SE); ^3^ P-values represent differences between 2015–2016 and 1988–1994; ^4^ cycle trend beta denotes change from one time period to the next; ^5^ yearly trend beta denotes change per year; *p* < 0.01 deemed significant and in denoted in bold.

**Table 2:** Daily total, added, and natural phosphorus mean ^1,2^ intake per body weight (mg/kg) and percentage of total phosphorus as added for adults 19 years of age and older, the NHANES 1988–1994 and 2015–2016.

Subject Group	NHANES Cycle	Total Phosphorus	Added Phosphorus	Natural Phosphorus	% As Added
All (n = 55,202)	1988–1994	17.75 (0.14)	2.43 (0.04)	15.32 (0.13)	14.61 (0.21)
	2001–2002	17.31 (0.23)	2.51 (0.05)	14.80 (0.22)	15.20 (0.27)
	2003–2004	17.02 (0.18)	2.42 (0.04)	14.61 (0.18)	15.09 (0.21)
	2005–2006	17.42 (0.24)	2.34 (0.06)	15.08 (0.23)	14.22 (0.33)
	2007–2008	17.00 (0.25)	2.21 (0.04)	14.79 (0.24)	13.64 (0.28)
	2009–2010	17.97 (0.16)	2.30 (0.03)	15.67 (0.14)	13.22 (0.22)
	2011–2012	18.20 (0.20)	2.19 (0.05)	16.01 (0.19)	12.51 (0.25)
	2013–2014	17.83 (0.17)	2.22 (0.04)	15.61 (0.18)	12.98 (0.25)
	2015–2016	17.48 (0.21)	1.93 (0.04)	15.55 (0.21)	11.53 (.22)
	P ^3^	0.2859	**<0.0001**	0.3222	**<0.0001**
	Cycle Trend				
	Beta ^4^	0.06 (0.02)	−0.06 (0.01)	0.12 (0.02)	−0.43 (0.03)
	*p*-value	0.0163	**<0.0001**	**<0.0001**	**<0.0001**
	Yearly Trend				
	Beta ^5^	0.01 (0.01)	−0.02 (0.002)	0.03 (0.01)	−0.14 (0.01)
	*p*-value	0.0163	**<0.0001**	**<0.0001**	**<0.0001**
Males (n = 27,356)	1988–1994	19.44 (0.18)	2.63 (0.05)	16.80 (0.17)	14.42 (0.25)
	2001–2002	18.57 (0.35)	2.65 (0.08)	15.92 (0.33)	14.63 (0.32)
	2003–2004	18.36 (0.25)	2.57 (0.06)	15.79 (0.24)	14.75 (0.28)
	2005–2006	18.78 (0.29)	2.54 (0.08)	16.23 (0.29)	14.29 (0.35)
	2007–2008	18.30 (0.27)	2.33 (0.06)	15.96 (0.28)	13.39 (0.37)
	2009–2010	19.33 (0.24)	2.53 (0.06)	16.80 (0.22)	13.36 (0.33)
	2011–2012	19.78 (0.24)	2.36 (0.08)	17.42 (0.21)	12.36 (0.33)
	2013–2014	19.26 (0.26)	2.34 (0.07)	16.91 (0.24)	12.68 (0.29)
	2015–2016	18.75 (0.29)	2.03 (0.05)	16.73 (0.28)	11.42 (0.31)
	P ^3^	0.0447	**<0.0001**	0.8234	**<0.0001**
	Cycle Trend				
	Beta ^4^	0.05 (0.03)	−0.06 (0.01)	0.12 (0.03)	−0.40 (0.04)
	*p*-value	0.1227	**<0.0001**	**0.0005**	**<0.0001**
	Yearly Trend				
	Beta ^5^	0.01 (0.01)	−0.02 (0.003)	0.03 (0.01)	−0.14 (0.01)
	*p*-value	0.1227	**<0.00012**	**0.0006**	**<0.0001**
Females (n = 27,846)	1988–1994	16.12 (0.18)	2.24 (0.05)	13.88 (0.15)	14.80 (0.23)
	2001–2002	16.07 (0.25)	2.37 (0.07)	13.70 (0.23)	15.75 (0.41)
	2003–2004	15.72 (0.30)	2.26 (0.05)	13.46 (0.30)	15.42 (0.38)
	2005–2006	16.09 (0.35)	2.14 (0.07)	13.95 (0.33)	14.15 (0.35)
	2007–2008	15.76 (0.30)	2.08 (0.04)	13.68 (0.28)	13.86 (0.29)
	2009–2010	16.66 (0.20)	2.08 (0.04)	14.58 (0.19)	13.07 (0.25)
	2011–2012	16.67 (0.22)	2.02 (0.05)	14.65 (0.21)	12.65 (0.26)
	2013–2014	16.46 (0.21)	2.10 (0.06)	14.36 (0.21)	13.27 (0.34)
	2015–2016	16.24 (0.20)	1.83 (0.05)	14.41 (0.19)	11.63 (0.23)
	P ^3^	0.6450	**<0.0001**	0.0274	**<0.0001**
	Cycle Trend				
	Beta ^4^	0.07 (0.03)	−0.05 (0.01)	0.12 (0.03)	−0.45 (0.04)
	*p*-value	0.0156	**<0.0001**	**<0.0001**	**<0.0001**
	Yearly Trend				
	Beta ^5^	0.02 (0.01)	−0.02 (0.003)	0.04 (0.01)	−0.15 (0.01)
	*p*-value	0.0156	**<0.0001**	**<0.0001**	**<0.0001**

^1^ Results adjusted for age, gender, and ethnicity; ^2^ values are represented as mean (SE); ^3^ *p*-value represent differences between 2015–2016 and 1988–1994; ^4^ cycle trend beta denotes change from one time period to the next; ^5^ yearly trend beta denotes change per year; *p* < 0.01 deemed significant and denoted in bold.

**Table 3:** Top dietary sources ^1^ of total, natural, and added phosphorus intake (mg/day) and percentage of total phosphorus in adults (19+ years) by USDA food categories from the NHANES 2001–2016 (n = 39,796).

Food Category	Consumer N	Mean (SE), mg/day	Percent Daily (SE), %
Total Phosphorus			
Cheese	13,219	80.32 (1.65)	5.84 (0.11)
Pizza	3853	57.93 (1.64)	4.21 (0.12)
Chicken, whole pieces	8755	49.98 (1.30)	3.63 (0.10)
Milk, reduced fat	6495	46.09 (1.37)	3.35 (0.10)
Eggs and omelets	9057	42.70 (0.84)	3.10 (0.06)
Yeast breads	16,634	38.80 (0.63)	2.82 (0.04)
Cold cuts and cured meats	7455	37.60 (0.94)	2.73 (0.07)
Nuts and seeds	6480	37.10 (1.16)	2.70 (0.08)
Meat mixed dishes	4360	32.66 (0.93)	2.37 (0.07)
Burritos and tacos	2327	31.92 (1.61)	2.32 (0.11)
Beef, excludes ground	4218	29.68 (0.98)	2.16 (0.07)
Fish	3338	29.40 (1.26)	2.14 (0.09)
Milk, nonfat	2688	28.46 (1.46)	2.07 (0.10)
Natural Phosphorus			
Pizza	3853	57.93 (1.64)	4.84 (0.14)
Cheese	13,219	53.81 (1.11)	4.49 (0.09)
Milk, reduced fat	6495	46.09 (1.37)	3.85 (0.11)
Eggs and omelets	9057	42.70 (0.84)	3.56 (0.07)
Chicken, whole pieces	8755	42.40 (1.10)	3.54 (0.09)
Nuts and seeds	6480	37.10 (1.16)	3.10 (0.09)
Meat mixed dishes	4,360	32.54 (0.93)	2.72 (0.08)
Yeast breads	16,634	32.44 (0.54)	2.71 (0.04)
Burritos and tacos	2327	31.92 (1.61)	2.66 (0.13)
Beef, excludes ground	4218	29.68 (0.98)	2.48 (0.08)
Cold cuts and cured meats	7455	29.60 (0.76)	2.47 (0.06)
Milk, nonfat	2688	28.46 (1.46)	2.38 (0.12)
Fish	3338	28.13 (1.20)	2.35 (0.10)
Milk, whole	4684	26.65 (1.03)	2.22 (0.09)
Added Phosphorus			
Cheese	13,219	26.52 (0.56)	14.95 (0.29)
Soft drinks	14,356	16.60 (0.55)	9.36 (0.31)
Cakes and pies	5278	16.22 (0.44)	9.15 (0.23)
Rolls and buns	6998	10.79 (0.30)	6.08 (0.16)
Cookies and brownies	8830	10.60 (0.19)	5.98 (0.12)
Doughnuts, sweet rolls, pastries	4564	10.23 (0.28)	5.77 (0.15)
Tortillas	4770	10.06 (0.59)	5.67 (0.33)
Diet soft drinks	5299	9.53 (0.31)	5.37 (0.16)
Cold cuts and cured meats	7455	8.00 (0.19)	4.51 (0.10)
Chicken, whole pieces	8755	7.58 (0.20)	4.27 (0.11)
Yeast breads	16,634	6.36 (0.09)	3.58 (0.06)
Biscuits, muffins, quick breads	3611	5.84 (0.25)	3.30 (0.14)
Nutritional beverages	481	5.61 (0.51)	3.16 (0.28)
Cream and cream substitutes	8250	4.97 (0.17)	2.81 (0.10)
Pancakes, waffles, French toast	1925	4.60 (0.20)	2.59 (0.11)

^1^ Sources listed provide ≥2% of total phosphorus intake.

## Data Availability

The data presented in this study are from publicly available data in NHANES and other additional data are available in the article and supplementary material.

## Supplementary Materials

The following are available online at https://www.mdpi.com/article/10.3390/nu13072249/s1, Table S1: Range of phosphate ingredients used in foods, Table S2: Percentage of food category with a phosphate compound in the ingredient list.
